# Immune checkpoint inhibitors in the treatment of virus-associated cancers

**DOI:** 10.1186/s13045-019-0743-4

**Published:** 2019-06-10

**Authors:** Peipei Gao, Cordelle Lazare, Canhui Cao, Yifan Meng, Ping Wu, Wenhua Zhi, Shitong Lin, Juncheng Wei, Xiaoyuan Huang, Ling Xi, Gang Chen, Junbo Hu, Ding Ma, Peng Wu

**Affiliations:** 1 Cancer Biology Research Center (Key Laboratory of the Ministry of Education), Tongji Hospital, Tongji Medical College, Huazhong University of Science and Technology, Wuhan, Hubei China; 2 Department of Gynecologic Oncology, Tongji Hospital, Tongji Medical College, Huazhong University of Science and Technology, Wuhan, Hubei China

**Keywords:** Immunotherapy, Immune checkpoint inhibitors, Virus-associated cancers

## Abstract

Among all malignant tumors that threaten human health, virus-related tumors account for a large proportion. The treatment of these tumors is still an urgent problem to be resolved. The immune system is the “guard” of the human body, resisting the invasion of foreign substances such as viruses. Studies have shown that immunotherapy has clinical significance in the treatment of a variety of tumors. In particular, the emergence of immune checkpoint inhibitors (ICIs) in recent years has opened a new door to cancer therapy. Considering the potential role of ICIs in the treatment of virus-related cancers, we focused on their therapeutic effect in virus-associated cancers and explored whether the therapeutic effect in virus-associated cancers was related to virus infection status. Although there is no clear statistical significance indicates that ICIs are more effective in virus-associated cancers than non-virus infections, the efficacy of checkpoint inhibitors in the treatment of virus-related cancers is promising. We believe that this research provides a good direction for the implementation of individualized precision medicine.

## Background

Cancer is a major public health problem worldwide. According to the International Agency for Research on Cancer (IARC), there were about 18.1 million new cancer cases and 9.6 million cancer deaths in 2018 worldwide [1]. Carcinogenic viral infection is an important cause of cancer, especially in developing countries. Approximately 20% of all human cancers were attributable to carcinogenic viruses [2]. Seven viruses have been classified as well established carcinogenic viruses in human beings by the IARC [3]: human papillomavirus (HPV), hepatitis B virus (HBV), hepatitis C virus (HCV), Epstein-Barr virus (EBV), human herpesvirus type 8 (HHV-8, also known as Kaposi’s sarcoma herpesvirus), HIV type 1 (HIV-1), and human T cell lymphotropic virus type 1 (HTLV-1). Among them, the most important infectious viruses worldwide are HPV, HBV, HCV, and EBV.

Even though these carcinogenic viruses belong to different genus and use multiple mechanisms to promote cancer development, they may have several features in common [4, 5]. They have the ability to infect host cell and establish persistent infection. During this process, they have evolved strategies for virus replication and persistence, including evading the host immune surveillance, creating conditions for virus replication, and ensuring correct replication. More specifically, immune escape mechanisms include producing anti-inflammatory cytokines, inducing regulatory T (Treg) cells, and increasing the expression of immune checkpoint proteins.

Immune checkpoints mainly including programmed death 1 (PD-1) and cytotoxic T lymphocyte antigen 4 (CTLA-4) are membrane-bound molecules expressed on immune cells. Immune checkpoint inhibitors (ICIs) are predominantly monoclonal antibodies, which have shown to be effective in a variety of cancers [6–11]. They function to block the binding of immune checkpoint molecules to their ligands, reversing the inactivation of T cells, enhancing the immune response of T cells, and resisting foreign aggression such as virus infections. Theoretically, they could assist in virus clearance in infected patients and may have a greater effect in virus-associated cancers. Therefore, ICIs are of concern to us and may have special effects in virus-associated cancers.

The ICIs approved by the Food and Drug Administration (FDA) include anti-PD-1 (nivolumab and pembrolizumab), anti-PD-L1 (atezolizumab, avelumab, and durvalumab), and anti-CTLA-4 (ipilimumab and tremelimumab). There are a series of clinical trials involving the efficacy of ICIs in virus-related cancers. Published clinical trials evaluating the efficacy of ICIs were summarized in Table 1, and the ongoing clinical trials were listed in Table 2.

**Table 1:** Published clinical trials evaluating ICIs in virus-related cancers

Viruses	ICIs	Cancers	Response rates	Ref.
HPV	Pembrolizumab	R/M cervical cancer	17%	[12]
	Nivolumab	GYN cancers	20.8%	[13]
	Nivolumab	SCCA	24%	[14]
	Pembrolizumab	R/M HNSCC	18%	[15]
		HPV-positive	25%	
		HPV-negative	14%	
	Durvalumab	R/M HNSCC	16.2%	[16]
		HPV-positive	29.4%	
		HPV-negative	10.8%	
HBV/HCV	Nivolumab	HCV infection	11.1%	[17]
	Tremelimumab	HCC	17.6%	[18]
	Tremelimumab	HCC	26.3%	[19]
	Nivolumab	HCC	15%-20%	[20]
		HBV/HCV-positive	14-20%	
		HBV/HCV-negative	21-23%	
EBV	Nivolumab	R/M NPC	20.5%	[21]
	Pembrolizumab	R/M NPC	25.9%	[22]
	Pembrolizumab	NK/T cell lymphoma	71.4%	[23]
	Pembrolizumab	NHL	23.3%	[24]
	Pembrolizumab	EBV-positive GC	100%	[25]
	Nivolumab	EBV-negative GC	25%	[26]
HIV	Nivolumab	Cancer (HIV-positive)	27%-63%	[27]
	Ipilimumab			
HTLV-1		Adult T cell lymphoma		
HHV-8		Kaposi’s sarcoma		

*Abbreviations*: *ICIs* immune checkpoint inhibitors, *HPV* human papillomavirus, *HBV* hepatitis B virus, *HCV* hepatitis C virus, *EBV* Epstein-Barr virus, *HHV-8* human herpesvirus type 8, *HTLV-1* human T cell lymphotropic virus type 1, *R/M* recurrent and/or metastatic, *GYN cancers* cervical, vaginal, and vulvar cancers, *SCCA* squamous cell carcinoma of the anal canal, *HNSCC* head and neck squamous cell carcinoma, *HCC* hepatocellular carcinoma, *NPC* nasopharyngeal carcinoma, *GC* gastric cancer, *NHL* non-Hodgkin lymphoma

**Table 2:** The ongoing clinical trials of ICIs in virus-associated cancers

Trial identifier	Phase	Study title	Treatment	Patients
ICIs in HPV-associated cancers				
NCT02054806	I	Pembrolizumab (MK-3475) in participants with advanced solid tumors (KEYNOTE-28)	Pembrolizumab	Advanced solid tumors
NCT01848834	I	Pembrolizumab (MK-3475) in participants with advanced solid tumors (KEYNOTE-012)	Pembrolizumab	Advanced Solid Tumors
NCT02488759	I/II	Nivolumab, and nivolumab Combination Therapy in Virus-associated Tumors (CheckMate 358)	Nivolumab Ipilimumab	Virus-associated tumors
NCT02314169	II	Nivolumab with or without ipilimumab in treating patients with refractory metastatic anal canal cancer (NCI 9673)	Ipilimumab Nivolumab	R/M SCCA
NCT02105636	III	Trial of nivolumab vs therapy of investigator’s choice in recurrent or metastatic head and neck carcinoma (CheckMate 141)	Nivolumab	R/M HNC
NCT02379520	I	HPV-16/18 E6/E7-specific T lymphocytes in patients with relapsed HPV-associated cancers	Nivolumab	HPV-related cancers
NCT03841110	I	FT500 as monotherapy and in combination with immune checkpoint inhibitors in subjects with advanced solid tumors	Nivolumab Pembrolizumab Atezolizumab	Advanced solid tumors
NCT03228667	II	QUILT-3.055: ALT-803 in combination with PD-1/PD-L1 checkpoint inhibitor in patients with advanced cancer	Pembrolizumab Nivolumab Atezolizumab Avelumab	Advanced cancers
NCT02890368	I	Intratumoral injections of TTI-621 in subjects with relapsed and refractory solid tumors and mycosis fungoides	PD-1/PD-L1 Inhibitor	HPV-related malignant neoplasm
NCT03735290	I/II	ILIxadencel administered into tumors in combination with checkpoint inhibitor (CPI) in patients with advanced cancer (ILIAD)	Pembrolizumab	Advanced cancer
NCT02632344	II	Pembrolizumab for HPV-associated recurrent respiratory papilloma patients with laryngeal, tracheal, and/or pulmonary involvement	Pembrolizumab	HPV-associated papilloma patients
NCT01693783	II	Ipilimumab in treating patients with metastatic or recurrent human papilloma virus-related cervical cancer	Ipilimumab	R/M HPV-related cervical cancer
ICIs in HBV/HCV-associated cancers				
NCT01853618	I	Tremelimumab with chemoembolization or ablation for liver cancer	Tremelimumab	Liver cancer
NCT01658878	I/II	Nivolumab or nivolumab in combination with other agents in patients with advanced liver cancer (CheckMate040)	Nivolumab Ipilimumab	Advanced liver cancer
NCT03841110	I	FT500 as monotherapy and in combination with ICIs in subjects with advanced solid tumors	FT500 Nivolumab Pembrolizumab Atezolizumab	Advanced solid tumors (HCC)
NCT03228667	II	QUILT-3.055: a study of ALT-803 in combination with PD-1/PD-L1 checkpoint inhibitor in patients with advanced cancer	Pembrolizumab Nivolumab Atezolizumab Avelumab	Advanced cancer
NCT03419481	II	Pembrolizumab in patients with HBV-related HCC	Pembrolizumab	HBV-related HCC
NCT02402699	II	Ipilimumab 60-month pharmacovigilance protocol for advanced melanoma patients who are hepatitis B and/or hepatitis C virus positive in Taiwan (Yervoy RMP)	Ipilimumab	Melanoma (HBV/HCV positive)
ICIs in EBV-associated cancers				
NCT02339558	II	Nivolumab in treating patients with recurrent and/or metastatic NPC	Nivolumab	R/M NPC
NCT02054806	I	Study of pembrolizumab (MK-3475) in participants with advanced solid tumors (MK-3475-028/KEYNOTE-28)	Pembrolizumab	Advanced solid tumors
NCT01848834	I	Study of pembrolizumab (MK-3475) in participants with advanced solid tumors (MK-3475-012/KEYNOTE-012)	Pembrolizumab	Advanced solid tumors
NCT02488759	I/II	An investigational immuno-therapy study to investigate the safety and effectiveness of nivolumab, and nivolumab combination therapy in virus-associated tumors (CheckMate358)	Nivolumab Ipilimumab	Virus-associated tumors
NCT03258567	II	Nivolumab in EBV-positive lymphoproliferative disorders and EBV-positive NHL	Nivolumab	EBV-positive lymphoproliferative disorders EBV-positive NHL
NCT02973113	I	Combining nivolumab with Epstein-Barr virus-specific T cells (EBVSTS) in relapsed/refractory EBV-positive lymphoma patients (PREVALE)	Nivolumab	EBV-positive lymphoma
NCT03038672	II	Nivolumab with or without varlilumab in treating patients with relapsed or refractory aggressive B cell lymphomas	Nivolumab Varlilumab	Aggressive B cell lymphomas (EBV-positive)
NCT03015896	I/II	Nivolumab and lenalidomide in treating patients with relapsed or refractory non-Hodgkin or Hodgkin lymphoma	Nivolumab	NHL or HL
NCT03267498	II	Nivolumab + chemoradiation in stage II–IVB nasopharyngeal carcinoma (NPC)	Nivolumab Chemoradiation	NPC
NCT02834013	II	Nivolumab and ipilimumab in treating patients with rare tumors	Nivolumab Ipilimumab	NPC
NCT03427827	III	Adjuvant PD-1 antibody in locoregionally advanced NPC after chemoradiotherapy	PD-1 antibody	NPC
NCT03390738	II	Nivolumab as treatment for recurrent/metastatic NPC after failing 2 lines or more previous chemotherapy	Nivolumab	R/M NPC
NCT03769467	I/II	Tabelecleucel in combination with pembrolizumab in subjects with EBV+ NPC (ATA129-NPC-202)	Pembrolizumab	EBV-positive NPC
NCT03586024	I/II	Pembrolizumab in patients with relapsed or refractory extranodal NK/T cell lymphoma (ENKTL), nasal type, and EBV-associated diffuse large B cell lymphomas	Pembrolizumab	EBV-associated diffuse large B cell lymphomas
NCT03257163	II	Pembrolizumab, capecitabine, and radiation therapy in treating patients with mismatch repair-deficient and EBV-positive gastric cancer	Pembrolizumab	EBV-positive GC
NCT03544099	II	Pembrolizumab for nasopharyngeal carcinoma patients with detectable plasma Epstein-Barr virus DNA	Pembrolizumab	EBV-positive NPC
NCT03813394	I/II	Bevacizumab and pembrolizumab combination in EBER-ISH positive NPC (2018/00947)	Pembrolizumab	NPC
NCT03160079	I/II	Blinatumomab and pembrolizumab for adults with relapsed/refractory B cell acute lymphoblastic leukemia with high marrow lymphoblasts	Pembrolizumab	Relapsed/refractory B cell ALL
NCT02950220	I	Pembrolizumab and ibrutinib in treating patients with relapsed or refractory non-Hodgkin lymphoma	Pembrolizumab	Relapsed or refractory NHL
NCT03491345	II	K-basket, avelumab, biomarker-driven, advanced solid tumor	Avelumab	EBV-positive mutation tumor
NCT02875613	II	Avelumab for recurrent/metastatic nasopharyngeal cancer	Avelumab	R/M NPC
NCT03735290	I/II	Evaluate the safety and effectiveness of ILIxadencel administered into tumors in combination with checkpoint inhibitor (CPI) in patients with advanced cancer	Pembrolizumab	Advanced cancer
ICIs in virus-associated cancers (HIV, HTLV, HHV-8)				
NCT02408861	I	Ipilimumab and nivolumab in advanced HIV-associated solid tumors with expansion cohorts in HIV-associated solid tumors and a cohort of HIV-associated classical Hodgkin lymphoma	Nivolumab Ipilimumab	Advanced HIV-associated solid tumors
NCT03316274	I	Evaluate the safety, feasibility, and immunologic correlatives of intra-lesional nivolumab therapy for limited cutaneous Kaposi sarcoma	Nivolumab	Limited cutaneous Kaposi sarcoma
NCT03367754	I	A single dose of pembrolizumab in HIV-infected people	Pembrolizumab	HIV-infected people
NCT02595866	I	Pembrolizumab in treating patients with HIV and relapsed, refractory, or disseminated malignant neoplasms	Pembrolizumab	Patients with HIV and malignant neoplasms
NCT03239899	I	PD-1 inhibition to determine CNS reservoir of HIV-infection	Pembrolizumab	HIV infection
NCT03767465	Observational	Treatment with ICIs of HIV-infected subjects with cancer (PembroHIV)	ICIs	HIV-infected subjects with cancer
NCT03075553	II	Nivolumab in treating patients with relapsed or refractory peripheral T cell lymphoma	Nivolumab	Relapsed or refractory peripheral T cell lymphoma
NCT02631746	II	Nivolumab in treating patients with HTLV-associated T cell leukemia/lymphoma	Nivolumab	HTLV-associated T cell leukemia/lymphoma
NCT03469804	II	Phase II multicentric study of pembrolizumab in classic or endemic Kaposi’s sarcoma	Pembrolizumab	Classic or endemic Kaposi’s sarcoma
NCT03038672	II	Nivolumab with or without varlilumab in treating patients with relapsed or refractory aggressive B cell lymphomas	Nivolumab Varlilumab	Relapsed or refractory aggressive B cell lymphomas
NCT03219671	II	Nivolumab and ipilimumab in classical Kaposi sarcoma	Nivolumab Ipilimumab	Classic Kaposi sarcoma

*Abbreviations*: *ICIs* immune checkpoint inhibitors, *HPV* human papillomavirus, *HBV* hepatitis B virus, *HCV* hepatitis C virus, *EBV* Epstein-Barr virus, *HHV-8* human herpesvirus type 8, *HTLV-1* human T cell lymphotropic virus type 1, *R/M* recurrent and/or metastatic, *GYN cancers* cervical, vaginal, and vulvar cancers, *SCCA* squamous cell carcinoma of the anal canal, *HCC* hepatocellular carcinoma, *NPC* nasopharyngeal carcinoma, *GC* gastric cancer, *NHL* non-Hodgkin lymphoma

### HPV-associated cancers

Human papillomavirus (HPV) is a circular DNA virus, which infects the genital mucosa, the oral mucosa, and the basal keratinocytes of the skin, mainly spreading by sexual contact. It was reported that HPV caused more than half of all infection-attributable cancers in women worldwide [3]. It is a common pathogen of cancers including cervix, vaginal, vulvar, anal, penile, and oropharyngeal cancers [28]. Although the incidence rate of cervical cancer in developed countries has been declining due to cancer screening programs and vaccination programs, the incidence rates of HPV-associated oropharyngeal, anal, and vulvar cancers increased from 2000 to 2009 [29]. Despite the wide use of multiple treatment options, most HPV-related cancers are still difficult to cure around the world. According to the National Comprehensive Cancer Network (NCCN) Clinical Practice Guidelines in Oncology, surgical resection is the treatment of choice for early stage cancers and chemo-radiotherapy is the standard treatment for locally advanced diseases. However, traditional treatment options such as systemic chemotherapy, surgery, and radiotherapy have limited effect for patients who have recurrent or metastatic cancers. The emergence of ICIs in recent years provides new hope for the treatment of these cancers.

In 2018, the FDA approved pembrolizumab for recurrent or metastatic cervical cancer based on results from the Keynote-028 trial [12], providing a new treatment option for cervical cancer. In this study, the overall response rate (ORR) was 17% (95% CI 5–37%). However, the HPV infection status of patients was not clear. A study (CheckMate358/NCT02488759) exploring the safety and efficacy of nivolumab in virus-associated cancers is being undertaken. In CheckMate358 [13], adults with cervical, vaginal, and vulvar cancers were eligible to receive nivolumab until progression or unacceptable toxicity. Of 24 treated patients, ORR was 20.8% and disease control rate (CR + PR + SD) was 70.8% at a median follow-up of 31 weeks. All responses were in patients with cervical cancer and were observed independent of HPV status. Nivolumab has shown encouraging therapeutic effect in patients with cervical cancer and is worthy of further evaluation in these patients.

Squamous cell carcinoma of the anal canal (SCCA) is an uncommon malignancy associated with HPV infection. The treatment of anal cancer depends on accurate staging, and chemo-radiotherapy is the main treatment for most patients [30]. A single-arm, multicenter, phase II trial (NCI-9673/NCT02314169) studied the therapeutic effect of nivolumab in patients with metastatic SCCA [14]. Among the 37 patients who were enrolled and received nivolumab, the response rate was 24% (95% CI 15–33). Given the high prevalence of HPV in SCCA and HPV was detected in all tested specimens in this study, the interaction of HPV with the tumor microenvironment could be responsible for the immune response.

Head and neck squamous cell carcinoma (HNSCC) comprises the majority of head and neck cancers and represents a heterogeneous group of tumors that arise from the squamous epithelium of the oral cavity, oropharynx, larynx, and hypopharynx [31]. In addition to the established risk factors such as smoking and alcohol consumption, HPV infection has become an important factor in the epidemiology and prognosis of HNSCC, mainly in oropharyngeal cancer [32, 33]. The most recent development in the treatment of HNSCC is immunotherapy. A clinical trial (Keynote-012), which evaluated the safety and antitumor activity of pembrolizumab in patients with recurrent or metastatic (R/M) HNSCC, published results recently [15]. Sixty patients with HNSCC were enrolled and treated: 23 (38%) patients were HPV-positive and 37 (62%) were HPV-negative. The proportion of patients with an overall response by central imaging review was 18% (95% CI 8–32) in all patients, 25% (95% CI 7–52) in HPV-positive patients, and 14% (95% CI 4–32) in HPV-negative patients. Another clinical trial (NCT02207530) is an international, multi-institutional, single-arm study [16], which evaluated durvalumab in patients with platinum-refractory R/M HNSCC. Among evaluable patients, ORR was 16.2% (95% CI 9.9–24.4), 29.4% (95% CI 15.1–47.5) among HPV-positive patients, and 10.8% (95% CI 4.4–20.9) in HPV-negative patients. Therefore, in HPV-positive HNSCC, the treatment of ICIs was seemingly more effective. This is consistent with previous reports that biological features of HPV-related head and neck cancers contribute to improved response [34–36]. In a systematic review evaluating the efficacy of ICIs on HNSCC [37], five studies analyzing the OS or the PFS stratified according to HPV-status were included. Four [15, 38–40] of the five studies demonstrated a higher OS or PFS in HPV-positive patients compared to HPV-negative patients. One study [41] found no difference in OS and PFS between the two subgroups.

### HBV or HCV-related hepatocellular carcinoma

Hepatocellular carcinoma (HCC) is one of the most frequently occurring cancers in the world and ranks third in global incidence [1]. HCC usually occurs in the setting of chronic liver inflammation and is mainly induced by viral hepatitis infection (HBV or HCV). Treatment of HCC including surgical resection, liver transplantation, and systemic therapy varies based on the stage of disease. However, only a small number of patients are suitable for surgical resection due to the extent of disease or poor liver function, and systemic treatment with sorafenib has displayed a comparatively modest role [42]. Therefore, there is an urgent need for new and better systemic therapy for HCC.

In a randomized, double-blind, placebo-controlled study (NCT00703469), the antivirus potential of BMS-936558 (MDX-1106/nivolumab) was explored in patients with chronic HCV infection [17]. Of the 54 patients who were treated (45 BMS-936558, nine placebo), clinical response (serum HCV RNA decline at least two consecutive visits) was observed in six patients (five BMS-936558, one placebo). There was no significant difference in clinical response rates between the nivolumab group (11.1%) and the placebo group (11.1%). Another clinical trial (NCT01008358) was to evaluate the antitumor and antivirus effect of tremelimumab in patients with HCC and chronic HCV infection [18]. Twenty patients were assessable for toxicity and virus response, and 17 were assessable for tumor response. As a result, the partial response rate was 17.6% and disease control rate was 76.4%. A significant drop in virus load was observed. This antivirus effect was associated with an enhanced specific anti-HCV immune response, supporting further research on the anticancer effect of tremelimumab.

Studies have shown that the killing of tumors by direct methods (known as ablation) can result in the immune system being activated or switched on [19, 43]. ICIs may enhance this effect of anticancer therapy by activating the immune system to recognize and kill residual cancer lesions. Here, a study (NCT01853618) aimed to demonstrate that whether tremelimumab could be combined with ablation safely and feasibly [19]. Thirty-two patients with HCC were enrolled. Five of 19 evaluable patients (26.3%) achieved a confirmed partial response, and 12 of 14 patients (85.7%) with quantifiable HCV experienced a marked reduction in virus load. Therefore, tremelimumab in combination with tumor ablation is a potential new treatment for patients with advanced HCC, leading to the accumulation of CD8+ T cells and the reduction in HCV virus load.

In September 2017, FDA approved nivolumab for liver cancer as a second line treatment after failure of sorafenib based on the data of CheckMate040 [20]. In the multi-cohort trial, 262 adults with advanced HCC were treated. In the dose-escalation phase, cohorts included 23 patients without virus hepatitis and 25 patients with HCV or HBV infection. The ORR was 15% (95% CI 6–28) in the dose-escalation phase, including three complete responses and four partial responses. In the dose-expansion phase, 214 patients with advanced HCC were distributed in different cohorts: 56 patients were not infected with HCV or HBV and had not been treated with sorafenib previously or were intolerant, 57 had disease progression on sorafenib, and 101 patients were infected with HCV or HBV. An objective response was observed in 42 patients (20%; 95% CI 15–26) in the dose-expansion phase. The response rate across all cohorts was reported in 14–20% of HBV or HCV infected patients and 21–23% of uninfected patients. Disease control was seen in 55–66% of patients infected with HBV or HCV and 61–75% of patients without viral hepatitis. It seems that the therapeutic effect of ICIs was not significantly different between the cohort with viral infection and the cohort without viral infection.

### EBV-associated cancers

EBV, also called human gamma-herpesvirus 4 (HHV-4), causes chronic latent infection with lifelong persistence in about 95% of the world population [44]. It is associated with several kinds of human neoplasms, such as malignant lymphoma, nasopharyngeal carcinoma (NPC), and gastric cancer (GC).

EBV-associated NPC is one of the most common head and neck malignancies, and unfortunately, 70% of NPC patients have locally advanced disease at initial diagnosis. Nasopharyngectomy is one established treatment option for locally recurrent NPC [45]. Surgical procedures include traditional open methods, endoscopic nasopharyngectomy, and minimally invasive methods for nasopharyngeal resection using robotics. Radiotherapy alone and concurrent chemo-radiotherapy are important treatment approaches for NPC, but they have a limited effect on patients with locally advanced or distantly metastatic disease [46–48]. With an increasing understanding of the complex interaction between EBV, NPC, and the host immune system, ICIs appears to be a promising approach for the treatment of EBV-associated NPC [49].

A multinational study (NCI-9742) evaluated the antitumor activity of nivolumab in NPC [21]. In this study, patients with R/M NPC were treated with nivolumab until disease progression and plasma-based biomarkers were investigated. A total of 44 patients were evaluated and the ORR was 20.5%. There was no statistical correlation between ORR and plasma EBV DNA clearance. Even so, the promising result of nivolumab in R/M NPC has driven interest in exploring the use of ICIs in EBV-associated NPC. Another clinical trial (Keynote-028/NCT02054806) is a nonrandomized, multi-cohort trial of pembrolizumab in patients with PD-L1-positive advanced solid tumors [22]. Twenty-seven patients with R/M NPC received pembrolizumab up to two years or until disease progression or unacceptable toxicity. Partial response and stable disease were observed in seven and 14 patients, respectively. The ORR was 25.9% (95% CI 11.1–46.3) over a median follow-up of 20 months. However, the study did not clearly indicate the viral infection status of patients.

EBV, originally discovered through its association with Burkitt lymphoma, is etiologically linked to a wide range of lymphoproliferative lesions and malignant lymphomas of B, T, and NK cell origin [50]. In a study involving seven patients with relapsed or refractory NK/T cell lymphoma, pembrolizumab proved to be effective [23]. After a median of seven cycles of pembrolizumab and a follow-up of a median of six months, five patients (71.4%) achieved a complete response, with two having molecular remission (undetectable EBV DNA). This suggested that pembrolizumab was a potent strategy for NK/T cell lymphomas failing L-asparaginase regimens. Another clinical trial [24] published result recently, mainly comparing the efficacy of pembrolizumab between EBV-positive and EBV-negative relapsed or refractory non-Hodgkin lymphomas (NHL) in 30 patients. In this study, seven patients with EBV-positive NHL showed a response including NK/T cell lymphoma (44%) and primary mediastinal B cell lymphoma (25%), whereas EBV-negative subtypes such as diffuse large B cell lymphoma and T-lymphoblastic lymphoma did not respond. In addition, high PD-L1 expression (positive staining > 50% of tumor cells) was found in NK/T cell lymphoma and primary mediastinal B cell lymphoma than other subtypes. Thus, PD-L1 expression was significantly higher (*p* < 0.001) in EBV-positive (56%) than EBV-negative NHL (11%). It is anticipated that the better therapeutic effect of ICIs in EBV-positive lymphoma may be related to high expression of PD-L1.

In addition, EBV-positive gastric cancer (GC) is also under our consideration. Most recently, extremely high ORR (100%) of pembrolizumab was reported in six patients with EBV-positive metastatic GC [25]. However, another study evaluated the effect of nivolumab showing that 25% EBV-positive advanced GC achieved an objective response [26]. Therefore, EBV status as a predictor of treatment outcome should be evaluated in a larger cohort.

### Other carcinogenic viruses

Other viruses that induce cancers include HIV, HTLV-1, and HHV-8. Among them, HIV is special in attributable risk calculations because its increased cancer risk only combine with other carcinogenic infectious factors [51]. A systematic review [27] was conducted to summarize the efficacy of ICIs therapy in HIV-positive cancers. Among 34 patients with known paired pretreatment and posttreatment HIV loads, HIV remained suppressed in 26 of the 28 (93%) with undetectable HIV load. ORR in these HIV-related patients was 30% for non-small cell lung cancer, 27% for melanoma, and 63% for Kaposi sarcoma. Therefore, ICIs may be an effective treatment option in this patient population. There are several clinical trials to assess the safety and efficacy of ICIs in HIV-infected patients. A placebo-controlled, dose-escalating study (NCT02028403) of BMS-936559 (anti-PD-L1 antibody) was conducted in HIV-1-infected adults [52]. The plasma HIV-1 RNA was detected by a single-copy assay. Of six men who received BMS-936559, the mean percentage of HIV-1 Gag-specific CD8+ T cells increased in two participants, illustrating single BMS-936559 infusions appeared to enhance HIV-1-specific immunity in participants. An open-label, multiple ascending dose study (NCT03407105) assessed the safety of ipilimumab and whether ipilimumab enhanced the immune response to HIV-1 in HIV-1-infected participants [53]. In this study, two participants (8.3%) had a decrease from baseline HIV-1 RNA, while 14 participants (58.3%) had an increase from baseline HIV-1 RNA. Ipilimumab was well tolerated and was associated with variations in HIV-1 RNA. However, the mechanisms underlying the increased variation in HIV-1 RNA is unclear and needs further study.

In addition, HTLV-1 is a retrovirus of the human T-lymphotropic virus family that has been related with several kinds of diseases including aggressive adult T cell lymphoma (ATL) and HTLV-1-associated myelopathy [54]. HHV-8 is also known as Kaposi’s sarcoma-associated herpesvirus (KSHV) and causes Kaposi sarcoma commonly occurring with acquired immune deficiency syndrome (AIDS). Clinical trials of these viruses were also listed in Table 2.

## Conclusions

At present, immunotherapy is widely use clinically, but is not always effective. It is not wise to administer immunotherapy without knowing the genetic background of the patient [55]. We need to screen out biomarkers to anchor which person is suitable for the application of checkpoint inhibitors. Future studies should focus on identifying biomarkers, such as virus infection status, to improve patient selection and help predict response. This article focuses on patients with viral-associated cancers and explores the therapeutic effects of ICIs.

In virus-induced cancers, the mechanisms by which viruses induce cancers are different, producing different mutation loads in tumors [44, 56–58]. Carcinogenic virus devastates host cellular structure, resulting in the engagement of virus DNA and host cell factors and the induction of DNA damage response (DDR). DDR increases their mutational rate, accelerates host chromosomal alteration, and as a consequence, facilitates virus replication [59, 60]. EBV and HPV are two examples that promote DDR and activate mutation. It has been reported that the mutation load determines the sensitivity of the tumor to PD-1 blockade [61]. Therefore, ICIs have different therapeutic effects on virus-related cancers.

In addition, the interplay of immune checkpoints and their ligands is complex, occurring at different stages of T cell activation and function. Similarly, they work at different stages of tumorigenesis [62]. In virus-associated cancers, expression of viral oncoproteins makes these tumors an effective target for ICIs. PD-1 is a T cell co-inhibitory receptor, which is expressed on multiple immune cells, including T cells, B cells, natural killer cells, dendritic cells, and monocytes. When it binds to its ligands, PD-L1 or PD-L2, the activation and differentiation of cytotoxic T cell are both downregulated [63]. PD-L1 expression levels are found to be increased in various cancers, providing an additional pathway for immune evasion by inactivation of T cells [64–69]. On the other hand, PD-L1 expression is a controversial prognostic factor in different preclinical trials evaluating the potential role of ICIs in cancers. Multiple studies have shown that PD-L1 overexpression is present in virus-infected cancers compared to non-virus-infected cancers [70–72]. For example, EBV-induced NPC expresses higher levels of PD-L1 compare to EBV-negative nasopharyngeal carcinoma [70]. Given the abundant evidence for immune exhaustion in chronic virus infections and virus-associated malignancies [62, 73], anti-PD-L1 blockers deserve to be investigated as a therapeutic strategy in virus-associated cancers.

Through the above clinical trials, there is no clear statistical significance indicates whether ICIs are more effective in virus-infected population than non-virus infections. However, the efficacy of ICIs in treating virus-associated cancers is rather promising. These virus-induced cancers present a specific immunological profile that virus-positive cancers often exhibit increased infiltration of cytolytic cell types compared to their virus-negative counterparts, and their responses to ICIs are expected to be different from other cancers [4]. More clinical trials are needed to evaluate the value of viral infections as a predictive factor in treatment based on checkpoint inhibitors. We can expect that if more clinical trial results are published, the efficacy of ICIs in virus-associated cancers will be highlighted and better application prospects will be developed.

## Data Availability

Not applicable.

## Abbreviations

AIDS: Acquired immune deficiency syndrome
CI: Confidence interval
CTLA-4: Cytotoxic T lymphocyte antigen 4
DDR: DNA damage response
EBV: Epstein-Barr virus
FDA: Food and Drug Administration
HBV: Hepatitis B virus
HCC: Hepatocellular carcinoma
HCV: Hepatitis C virus
HHV-8: Human herpesvirus type 8
HNSCC: Head and neck squamous cell carcinoma
HPV: Human papillomavirus
HTLV-1: Human T cell lymphotropic virus type 1
IARC: International Agency for Research on Cancer
ICIs: Immune checkpoint inhibitors
KSHV: Kaposi's sarcoma-associated herpesvirus
NHL: Non-Hodgkin lymphomas
NPC: Nasopharyngeal carcinoma
ORR: Overall response rate
PD-1: Programmed death 1
R/M HNSCC: Recurrent or metastatic head and neck squamous cell carcinoma
SD: Stable disease
Treg: Regulatory T cell
